# Differential mRNA Expression Levels of Human Histone-Modifying Enzymes in Normal Karyotype B Cell Pediatric Acute Lymphoblastic Leukemia

**DOI:** 10.3390/ijms14023376

**Published:** 2013-02-06

**Authors:** Yan-Fang Tao, Li Pang, Xiao-Juan Du, Li-Chao Sun, Shao-Yan Hu, Jun Lu, Lan Cao, Wen-Li Zhao, Xing Feng, Jian Wang, Dong Wu, Na Wang, Jian Ni, Jian Pan

**Affiliations:** 1 Department of Hematology and Oncology, Children’s Hospital of Soochow University, Suzhou 215003, Jiangsu, China; E-Mails: taoyanfang1982@163.com (Y.-F.T.); pangyeli2011@163.com (L.P.); hsy139@126.com (S.-Y.H.); lujun_sz@yahoo.com.cn (J.L.); cl2012@sohu.com (L.C.); zhaowenli69@yahoo.com.cn (W.-L.Z.); xing_feng66@hotmail.com (X.F.); wj196312@vip.163.com (J.W.); wudong.1987@163.com (D.W.); ontheway120@126.com (N.W.); 2 Department of Gastroenterology, the 5th Hospital of Chinese PLA, Yinchuan 750004, Ningxia, China; E-Mail: du_xiaojuan123@163.com; 3 Department of Cell and Molecular Biology, Cancer Institute (Hospital), Chinese Academy of Medical Sciences, Peking Union Medical College, Beijing100021, China; E-Mail: sunlichao_1980@hotmail.com; 4 Translational Research Center, The Second Clinical School, Nanjing Medical University, Nanjing 210011, Jiangsu, China; E-Mail: ni_jian2008@163.com

**Keywords:** histone-modifying enzymes, pediatric acute lymphoblastic leukemia, real-time PCR array

## Abstract

Histone modification enzymes regulate gene expression by altering the accessibility of promoters to transcription factors. We sought to determine whether the genes encoding histone modification enzymes are dysregulated in pediatric acute lymphoblastic leukemia (ALL). A real-time PCR array was designed, tested and used to profile the expression of 85 genes encoding histone modification enzymes in bone marrow mononuclear cells from 30 pediatric ALL patients and 20 normal controls. The expression profile of histone-modifying genes was significantly different between normal karyotype B cell pediatric ALL and normal controls. Eleven genes were upregulated in pediatric ALL, including the histone deacetylases *HDAC2 and PAK1*, and seven genes were downregulated, including *PRMT2* and the putative tumor suppressor *EP300*. Future studies will seek to determine whether these genes serve as biomarkers of pediatric ALL. Ingenuity Pathway Analysis revealed that Gene Expression and Organ Morphology was the highest rated network, with 13 focus molecules (significance score = 35). Ingenuity Pathway Analysis also indicated that curcumin and miR-34 are upstream regulators of histone-modifying enzymes; future studies will seek to validate these results and examine the role of curcumin and miR-34 in leukemia. This study provides new clues into the molecular mechanisms of pediatric ALL.

## 1. Introduction

Acute lymphoblastic leukemia (ALL) is the most common malignancy diagnosed in children, representing nearly one third of all pediatric cancers [1–4]. Over the last decade, advances in the treatment of pediatric ALL have led to long-term event-free survival rates of approximately 80%. Despite the good overall prognosis, some of the less common subtypes of ALL have a high risk of relapse [1–4]. Rearrangements of the myeloid/lymphoid or mixed-lineage leukemia (*MLL*) gene at chromosome band 11q23 are detected in least 10% of cases and are associated with aggressive pediatric ALL. Unfortunately, young children with this genetic abnormality have a very poor prognosis and a survival rate of less than 20%, even after intensive therapy [5]. The *MLL* gene encodes a DNA-binding protein which methylates histone H3 lysine 4 (H3K4). MLL is a member of the group of histone-modifying enzymes, which is commonly disrupted in leukemia [6,7]. *MLL* translocations encode MLL fusion proteins which lack H3K4 methyltransferase activity, which results in abnormal histone modification [8,9].

Histone-modification provides an important regulatory platform for processes such as gene expression, DNA replication and repair, chromosome condensation and segregation and apoptosis. Disruption of these processes has been linked to the multistep process of carcinogenesis [10]. Alterations in histone-modifying enzymes can contribute to the development of a variety of human cancers. The new terminology “histone onco-modifications” has been proposed to describe the post-translational histone modifications linked to cancer [11,12]. Histones are the chief protein components of chromatin, acting as the spools around which DNA winds. Histones are no longer considered to be simple “DNA packaging” proteins, and are currently recognized to be regulators of chromatin dynamics. Histones are subject to a wide variety of post-translational modifications, including acetylation of lysines, methylation of lysines and arginines, serine and threonine phosphorylation, lysine ubiquitylation, glycosylation, sumoylation, adenosine diphosphate ribosylation and carbonylation, all of which are dynamically catalyzed by histone-modifying enzyme complexes [13,14]. Histone modifications influence chromatin-templated processes such as gene transcription, DNA repair and recombination. Histone lysine methylation and acetylation are enzymatically reversible processes which are “written” by lysine methyltransferases (KMTs) [15] and lysine acetyltransferases (KATs) [16], and “erased” by lysine demethylases (KDMs) [17–21] and histone deacetylases (HDACs) [22]. Overall, post-translational histone modifications provide an epigenetic mechanism for the regulation of a variety of normal and cancer-related processes. Growing evidence suggests that histone-modifying enzymes are dysregulated in human cancer. In fact, an extensive analysis of the expression patterns of histone-modifying enzymes could discriminate between tumor samples and their normal counterparts, and also cluster the tumor samples according to cell type [10]. However, little is currently known about the histone modification changes which occur during the development and progression of pediatric ALL.

Real-time PCR array systems are an ideal tool for analyzing the expression of a focused panel of genes [23]. The specificity of real-time PCR guarantees the amplification of a single gene-specific product in each reaction, allowing the expression level results to confidently reflect only the gene of interest. PCR arrays can determine the gene expression differences between two RNA samples, with results that are highly concordant with other quantitative gene expression analysis and microarray platforms. PCR arrays also deliver results comparable to high-density microarrays, as well as TaqMan Gene Expression Assays, a widely accepted method for validating the results of microarrays and other more complicated and expensive quantitative methods based on TaqMan assays [24]. In this study, we sought to analyze the mRNA expression profiles of histone-modifying enzymes in pediatric ALL using a powerful real-time PCR array platform.

## 2. Results and Discussion

### 2.1. Real-Time PCR Array Design

We designed and tested 88 real-time PCR primer pairs for quantitative gene expression analysis of genes involved in pediatric ALL. The primers for the target genes are listed in Supplementary File 1. Real-time PCR primers for histone-modifying enzymes. The human histone-modifying enzymes PCR array was designed to profile the expression of 85 key genes, which encode enzymes known or predicted to modify genomic DNA or histones to regulate chromatin accessibility, and therefore gene expression. *De novo* and maintenance DNA methyltransferases, and the enzymes responsible for the demethylation of CpG dinucleotides were represented on the array, NOTCH signaling, (*NOTCH1*, *NOTCH2*, *EP300*) and DNA methyltransferases (*DNMT1*, *DNMT3A*, *DNMT3B*). Enzymes catalyzing histone acetylation, methylation, phosphorylation, and ubiquitination were also included on the array, as well as deacetylases and demethylases. The genes included were the histone acetyltransferases: *ATF2*, *CDYL*, *CIITA*, *CSRP2BP*, *ESCO1*, *ESCO2*, *HAT1*, *KAT2A (GCN5L2)*, *KAT2B (PCAF)*, *KAT5 (HTATIP)*, *KAT8*, *KAT7*, *KAT6A*, *KAT6B*, *NCOA1*, *NCOA3*, *NCOA6*; histone methyltransferases: *CARM1 (PRMT4)*, *DOT1L*, *EHMT2*, *MLL*, *MLL3*, *PRMT1*, *PRMT2*, *PRMT3*, *PRMT5*, *PRMT6*, *PRMT7*, *PRMT8*, *SETDB2*, *SMYD3*; enzymes of histone phosphorylation: *AURKA*, *AURKB*, *AURKC*, *NEK6*, *PAK1*, *RPS6KA3*, *RPS6KA5*; enzymes of histone ubiquitination: *DZIP3*, *RNF2*, *RNF20*, *UBE2A*, *UBE2B*, *USP16*, *USP21*, *USP22*; DNA/histone demethylases: *KDM1A*, *KDM5B*, *KDM5C*, *KDM4A*, *KDM4C*, *MBD2*; and histone deacetylases: *HDAC1*, *HDAC2*, *HDAC3*, *HDAC4*, *HDAC5*, *HDAC6*, *HDAC7*, *HDAC8*, *HDAC9*, *HDAC10*, *HDAC11*. The array also included genes encoding *Drosophila* [S*u(var)*, E*(z)* and T*rithorax*] (SET) domain proteins, all of which contain a homologous domain that confers histone methyltransferase activity in some family members, SET Domain Proteins (Histone Methyltransferase Activity): *ASH1L*, *MLL3*, *MLL5*, *NSD1*, *SETD1A*, *SETD2*, *SETD3*, *SETD4*, *SETD6*, *SETD7*, *SETD8*, *SETDB1*, *SUV39H1*, *SUV420H1*, *WHSC1*.

### 2.2. Real-Time PCR Array Testing

**Figure 1:**
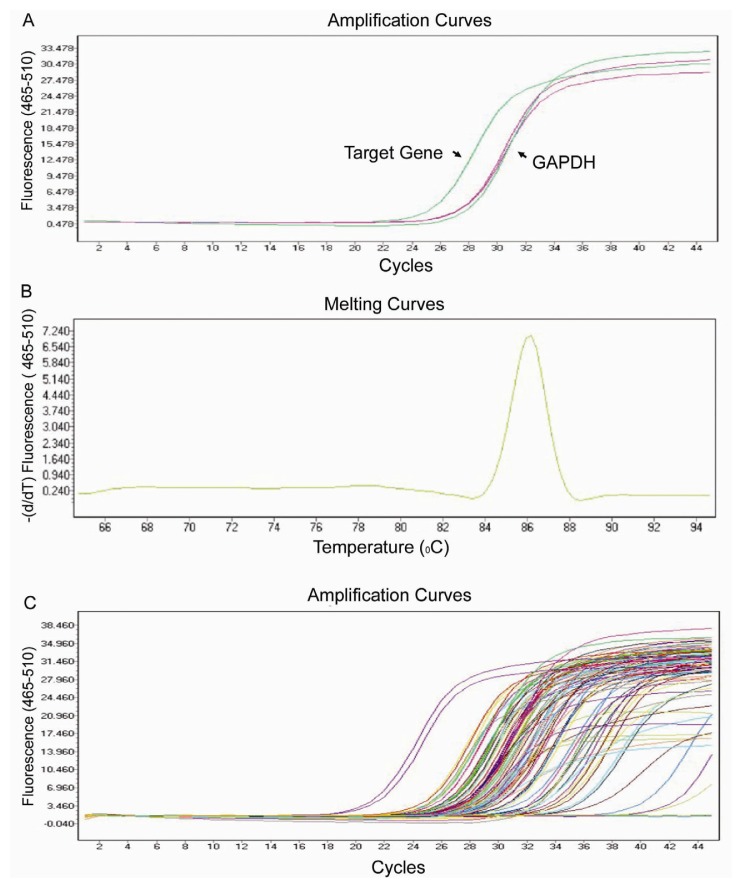
Design and testing of the real-time PCR array for human genes encoding epigenetic chromatin modification enzymes. (**A**) Amplification of a target gene and *GAPDH* in the real-time PCR array. Reactions were run on a Light cycler 480 (Roche, Basel, Switzerland) using universal thermal cycling parameters (95 °C for 5 min, 45 cycles of 10 s at 95 °C, 20 s at 60 °C and 15 s at 72 °C); (**B**) Melting curve analysis of the PCR product of a single target gene. Melting curves were generated using the parameters 10 s at 95 °C, 60 s at 60 °C, followed by continued melting; (**C**) Amplification of all of the genes in the PCR array.

Using real-time PCR, we can easily and reliably analyze the expression of a focused panel of genes involved in epigenetic chromatin modifications with this array. Each primer set was tested by expression analysis and melting curve analysis, to confirm that the primers were specific for the target gene (Figure 1). The flexibility, simplicity and convenience of standard SYBR Green PCR detection methodology makes PCR array systems accessible for routine use in any research laboratory.

### 2.3. Expression Profiling of Normal Karyotype B Cell Pediatric ALL and Normal Control Samples

**Figure 2:**
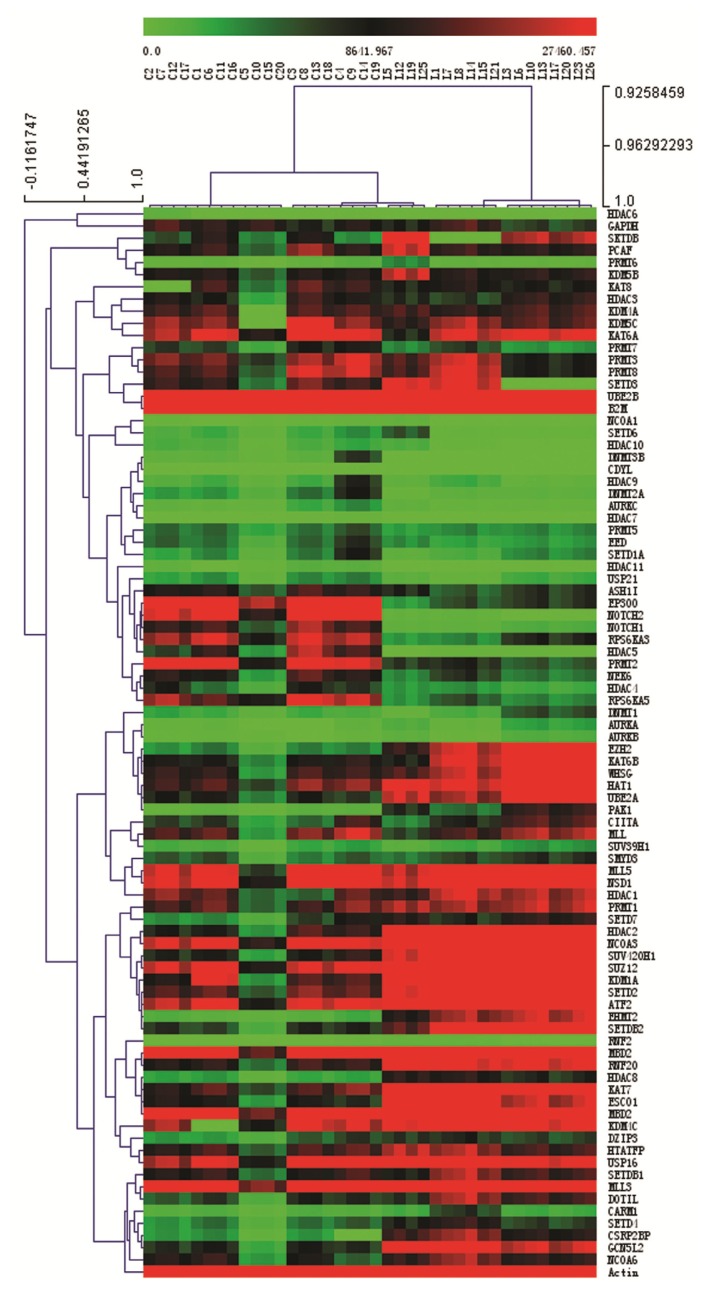
Expression and clustering analysis of differentially expressed genes encoding epigenetic chromatin modification enzymes in pediatric ALL and normal control samples. Clustering analysis of the gene expression data from the real-time PCR array. The comparative C_t_ method was used for quantification of gene expression. The gene expression levels for each target gene were normalized to the housekeeping gene *GAPDH* within the same sample (−ΔC_t_); then the relative expression of each gene (*n* = 87) was calculated using 10^6^ × Log_2_(−ΔC_t_). Gene expression in the normal karyotype B cell pediatric acute lymphoblastic leukemia (ALL) (*n* = 18) and control samples (*n* = 20) was analyzed using Multi Experiment View (MEV) clustering software.

**Figure 3:**
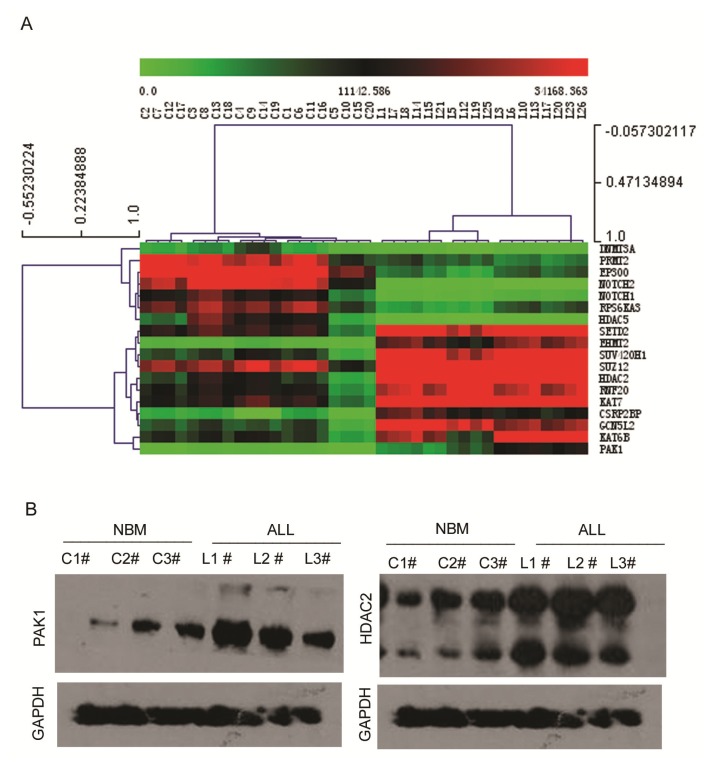
Expression and clustering analysis of differentially expressed genes encoding epigenetic chromatin modification enzymes in pediatric ALL and normal control samples. (**A**) The most significantly clustered genes between normal karyotype B cell ALL and normal controls; (**B**) Western-blot analysis the expression of PAK1 and HDAC2 in pediatric ALL and normal control samples.

We analyzed and clustered the gene expression profiles of bone marrow mononuclear cells from 30 pediatric ALL patients and 20 control samples using the real-time PCR array. The clinical features of the 30 pediatric ALL patients and 20 controls are listed in Table 1. We analyzed the original expression data using Multi Experiment View (MEV) clustering software. The cluster is not successful. This result showed pediatric ALL sample L2, L9, L28, L4, L16, L24, L11, L22, L27, L29 and L30 are different from other ALL samples. L2 and L9 are T cell ALL. L4 and L16 are B cell ALL with MLL fusion gene (Supplementary File 2. Cluster analysis the gene expression in 30 pediatric ALL patients and 20 controls samples). Heterogeneous genetic background may affect the expression of histone-modifying enzymes. So we do the second cluster, and we only clustered gene expression profiles from 18 normal karyotype B cell pediatric ALL patients and 20 control samples The gene expression profile in pediatric ALL was significantly different to the normal controls. Specific sets of genes clustered in normal karyotype B cell ALL (Figure 2). The most significantly clustered genes are shown in Figure 3A. The expression of PAK1 and HDAC2 between normal karyotype B cell ALL and normal control was certificated with western-blot (Figure 3B).

The gene expression profile in pediatric ALL was significantly different to the normal controls. Specific sets of genes clustered in normal karyotype B cell ALL (Figure 2). The most significantly clustered genes are shown in Figure 3A. The expression of PAK1 and HDAC2 between normal karyotype B cell ALL and normal control was certificated with western-blot (Figure 3B).

**Figure 4:**
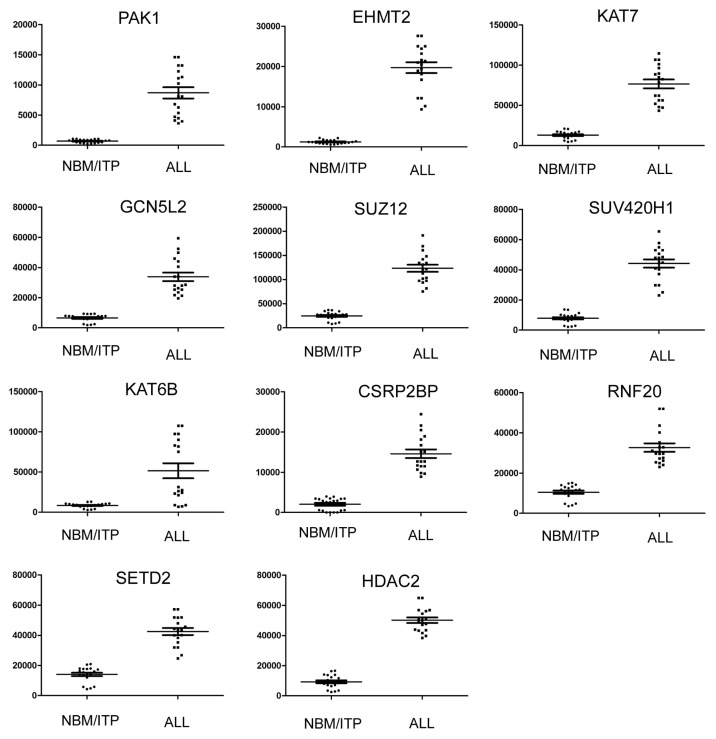
Expression of upregulated epigenetic chromatin modification genes in normal karyotype B cell pediatric ALL. Expression levels of the significantly upregulated genes in normal karyotype B cell pediatric ALL (*n* = 18), compared to the control samples (NBT/IPT; *n* = 20). Data is presented as the average ± SE; *p* values <0.05 were considered statistically significant.

The 11 genes upregulated in normal karyotype B cell pediatric ALL are listed in Table 2. The expression level of each upregulated gene in pediatric ALL is presented in Figure 4. Some of these upregulated genes have previously been studied in leukemia or other tumors. The gene expression profile of 12 HDAC genes was previously analyzed by quantitative real-time PCR in 94 consecutive cases of childhood ALL [10,25]. The ALL samples showed higher expression levels of *HDAC2*, compared to normal bone marrow samples [10,25], in agreement with this study. The epigenetic regulator *HDAC2* is often significantly overexpressed in solid tumors, can influence cell proliferation, apoptosis and differentiation, and has been suggested as a therapeutically important prognostic marker [26–29].

Changes in the levels and activity of p21 protein (Cdc42/Rac)-activated kinase 1 (PAK1) are also frequently described in human malignancies [30–33]. This phenomenon has been observed in various tumor types using a variety of techniques. The abnormalities reported include gene amplification, elevated mRNA and protein expression, and increased accumulation of the phosphorylated and, presumably, activated form of this enzyme. There are also intriguing observations regarding the accumulation of phosphorylated PAK1 specifically in the nuclei of malignant cells [34], which parallel the changes observed during tumor progression in a mouse model [35]. Importantly, elevated levels of PAK1 were identified to be an independent prognostic predictor of poor survival in ovarian cancer [36]. In breast cancer, nuclear expression of PAK1, in conjunction with phosphorylation of the estrogen receptor on the PAK1 site (serine 305), predicts resistance to tamoxifen therapy, and the cytoplasmic levels of PAK1 correlate with the recurrence rate and mortality [35,37]. Similarly, higher levels of PAK1 were associated with advanced tumor stage, metastasis and reduced survival in patients with gastric cancer [38,39]. There are also numerous reports of elevated PAK1 activity in cell lines, although in most of these cases such reports cannot rule out the possibility that the changes have been selected for or caused by *in vitro* culture.

**Figure 5:**
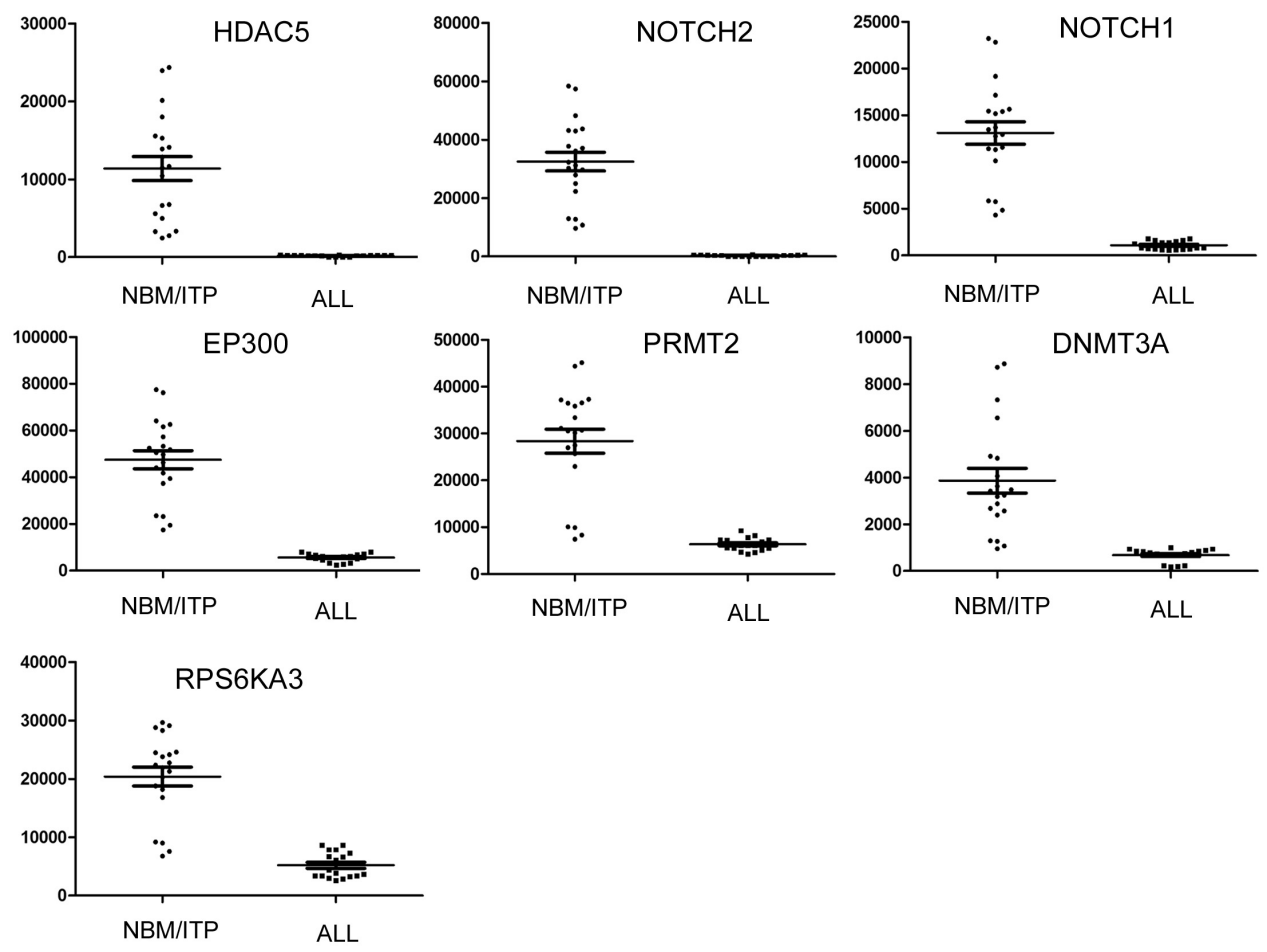
Expression of downregulated epigenetic chromatin modification genes in normal karyotype B cell pediatric ALL. Expression levels of the significantly downregulated genes in normal karyotype B cell pediatric ALL (*n* = 18), compared to the control samples (NBT/IPT; *n* = 20). Data is presented as the average ± SE; *p* values <0.05 were considered statistically significant.

The seven genes downregulated in pediatric ALL are listed in Table 3, and the expression level of each downregulated gene in pediatric ALL is presented in Figure 5. The putative tumor suppressor gene *EP300* is located on chromosome 22q13, a region which shows frequent loss of heterozygosity (LOH) in colon, breast and ovarian cancer. LOH across the *EP300* locus was detected in 38% of colon, 36% of breast, and 49% of ovarian primary tumors; however, no somatic mutations in *EP300* have been identified in any primary tumor [40,41]. EP300 is a histone acetyltransferase that regulates transcription via chromatin remodeling, and plays an important role in the processes of cell proliferation and differentiation. EP300 acetylation of tumor protein P53 (TP53) in response to DNA damage regulates the DNA-binding and transcription functions of TP53 [42]. The tumor suppressor gene protein arginine methyltransferase 2 (*PRMT2*) inhibits nuclear factor of kappa light polypeptide gene enhancer in B-cells 1 (NF-κB)-dependent transcription and promotes apoptosis, by blocking nuclear export of nuclear factor of kappa light polypeptide gene enhancer in B-cells inhibitor, alpha (IκB-α) via a leptomycin-sensitive pathway, which increases nuclear IκB-α accumulation and decreases NF-κB DNA binding [43–46]. The highly conserved *S*-adenosylmethionine-binding domain of PRMT2 mediates this effect. PRMT2 also renders cells susceptible to apoptosis induced by cytokines or cytotoxic drugs, most likely due to the effects of PRMT2 on NF-κB. Mouse embryo fibroblasts from *PRMT2* genetic knockouts have elevated NF-κB activity and decreased susceptibility to apoptosis, compared to wild-type or complemented cells. These data suggest that PRMT2 inhibits cell activation and promotes programmed cell death through a NF-κB-dependent mechanism.

### 2.4. Ingenuity Pathway Analysis of Dys-regulated Genes in Normal Karyotype B cell Pediatric ALL

**Figure 6:**
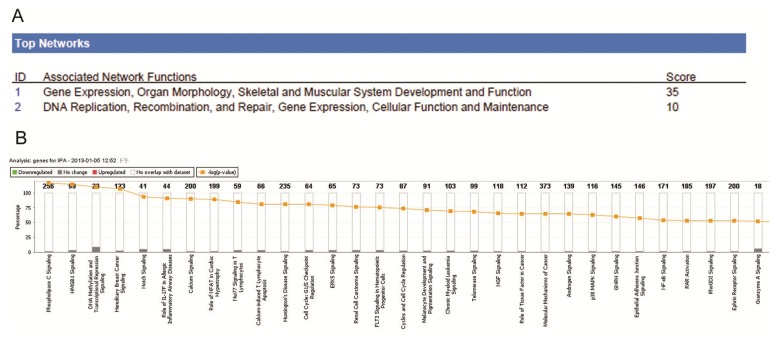

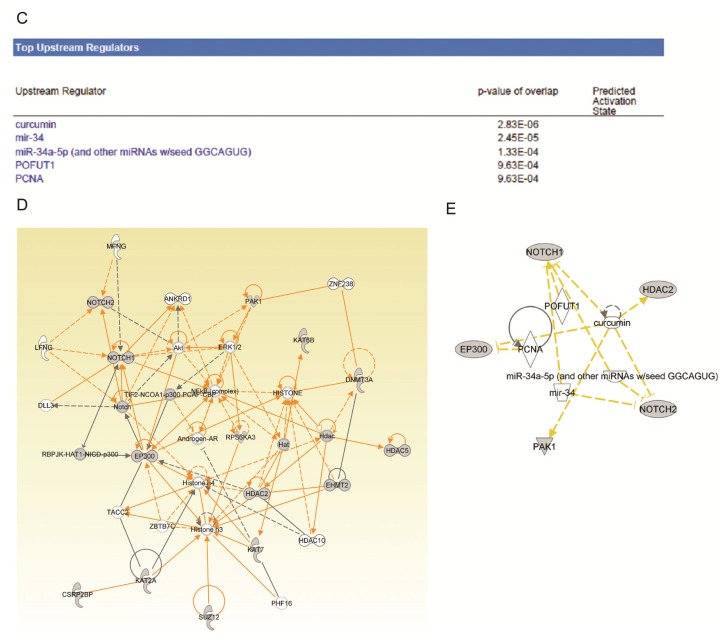
Summary of Ingenuity Pathway Analysis for dys-regulated epigenetic chromatin modification genes in normal karyotype B cell pediatric ALL. To investigate possible interactions between the differently regulated genes in pediatric ALL, datasets representing the 18 significantly altered genes were imported into the Ingenuity Pathway Analysis (IPA) Tool. (**A**) Top two networks obtained from IPA (with their respective scores) for the differently regulated genes in pediatric ALL; (**B**) Toxicology pathway list obtained from IPA analysis for the differently regulated genes in pediatric ALL. The *x*-axis represents the most significant toxicology functions based on the differentially expressed genes are highlighted; the *y*-axis represents the number of genes from the dataset that map to the pathway and the number of all known genes ascribed to the pathway. The yellow line represents the threshold *p* value (0.05), as calculated by Fisher’s test; (**C**) Upstream regulator list for the differently regulated genes in pediatric ALL. Curcumin and mir-34 were the two most significant upstream regulators of the differently regulated genes in pediatric ALL; (**D**) Network representation of the most highly rated network for the differently regulated genes in pediatric ALL. The shaded genes are statistically significant. Solid lines represent a direct interaction between two gene products, dotted lines represent indirect interactions; (**E**) Mapping of the genes associated with the upstream regulators for the differently regulated genes in pediatric ALL.

To investigate the possible biological interactions between the differently regulated genes in pediatric ALL, the datasets derived from the real-time PCR array analyses were imported into the Ingenuity Pathway Analysis (IPA) Tool. IPA analysis of the genes with a significantly altered expression profile in ALL revealed two significant networks (Figure 6A). Of these networks, Gene Expression and Organ Morphology was the highest rated network, with 13 focus molecules and a significance score of 35 (Figure 6D). The score is the probability that a collection of genes equal to or greater than the number of genes in the network could be achieved by chance alone. A score of 3 indicates a 1/1000 chance that the focus genes are not in the network due to random chance. The IPA analysis also grouped the differentially expressed genes in pediatric ALL into a number of other biological mechanisms related to Phospholipase C Signaling (1.79 × 10^−4^), HMGB1 Signaling (2.22 × 10^−4^), DNA methylation and Transcriptional Repression Signaling (2.99 × 10^−4^), Hereditary Breast Cancer Signaling (3.84 × 10^−4^) and Nocth Signaling (1.03 × 10^−3^, Figure 6B). Further results of the IPA analysis are provided in Supplementary File 3. IPA analysis of the significantly dys-regualted histone-modifying enzymes in pediatric ALL.

The IPA analysis also revealed that curcumin and mir-34 signaling were the two most important upstream regulators for the dysregulated histone-modifying enzymes in pediatric ALL, with *p* values of 2.83 × 10^−6^ and 2.45 × 10^−5^, respectively (Figure 6C). The genes associated with the upstream regulators are mapped in Figure 4E. Ectopic expression of miR-34 genes leads to marked effects on cell proliferation and survival, due to cell-cycle arrest in the G1 phase [47,48]. Interestingly, the introduction of miR-34a and miR-34b/c induced cellular senescence in primary human diploid fibroblasts, and overexpression of miR-34a induced apoptosis in tumor cells. MiR-34a has been shown to target and translationally repress sirtuin 1 (*SIRT1*) mRNA [49–51]. SIRT1, a histone-modifying enzyme, is a NAD-dependent deacetylase which has been shown to inhibit the activity of several pro-apoptotic proteins. Regulation of *SIRT1* by miR-34a forms part of a positive feedback loop which leads to enhanced activation of p53, once it has been initially activated. This study provides the first indication that other histone-modifying enzymes, in addition to *SIRT1*, may be dys-regulated by miR-34 in pediatric ALL.

The other upstream regulator of histone-modifying enzymes in normal karyotype B cell pediatric ALL revealed in this study was curcumin (diferuloylmethane), which is a polyphenol derived from the plant *Curcuma longa*, commonly known as turmeric. Recently, curcumin has been found to possess anti-cancer activity, as it exerts a number of effects on a variety of biological pathways involved in mutagenesis, oncogene expression, cell cycle regulation, apoptosis, tumorigenesis and metastasis. Curcumin has demonstrated anti-proliferative effects in multiple types of cancer, and is an inhibitor of the transcription factor NF-κB and its downstream gene products including *c-MYC*, *BCL-2*, *COX-2*, *NOS*, *Cyclin D1*, *TNF-α*, interleukins and *MMP-9*. In addition, curcumin affects a variety of growth factor receptors and cell adhesion molecules involved in tumor growth, angiogenesis and metastasis. Cultured leukemia cells are particularly responsive to curcumin [52–54]. As of 2011, more than 75 studies in peer-reviewed journals have reported that curcumin induces apoptosis and cell death in cultured animal and human leukemia cells. This study is the first to imply that curcumin may affect cancer cell growth and apoptosis via regulation of histone-modifying enzymes. In the future, we will seek to validate these results, and examine the role of curcumin and miR-34 in the molecular basis of leukemia.

## 3. Experimental Section

### 3.1. Patients and Samples

Bone marrow specimens were obtained at the time of diagnosis during routine clinical assessment of 30 patients with ALL, who presented at the Department of Hematology and Oncology, Children’s Hospital of Soochow University between 2010 and 2012. Ethical approval was provided by the Children’s Hospital of Soochow University Ethics Committee (No.SUEC2010-011 and No. SUEC2009-219-1), and informed consent was obtained from the parents or guardians. The main clinical and laboratory features of the patient cohort are summarized in Table 1. Additionally, bone marrow samples from 10 healthy donors from surgical operations and 10 patients with idiopathic thrombocytopenic purpura (ITP) were analyzed as controls. Bone marrow mononuclear cells (BMNCs) were isolated using Ficoll solution within 2 h after harvest.

### 3.2. RNA Extraction

BMNCs were immediately submerged in 4 mL TRIzol (Invitrogen, Carlsbad, CA, USA), and stored at −80 °C until further processing. A volume of 1.2 mL from each sample was centrifuged at 12,000*g* for 15 min at 4 °C to remove debris and DNA, then 1 mL of the supernatant was mixed with 200 μL chloroform, shaken for 15 seconds, incubated at RT for 2–3 min and centrifuged at 12,000*g* for 10 min at 4 °C. RNA was precipitated by adding 500 μL of the aqueous phase to an equal volume of isopropanol and centrifugation at 14,000*g* for 10 min at 4 °C. The RNA pellet was washed with 75% ethanol, centrifuged at 14,000*g* for 10 min at 4 °C, dried and resuspended in 60 μL DEPC-treated H_2_O. The final RNA concentration of the samples was determined using a spectrophotometer (Nanodrop 2000, Thermo-scientific, Wilmington, DE, USA) and the purity of the RNA samples was assessed by agarose gel electrophoresis.

### 3.3. Synthesis of cDNA

Synthesis of cDNA was performed using 4 μg of RNA in 10 μL reactions with SuperScript II reverse transcriptase (Invitrogen, Carlsbad, CA, USA), as recommended by the manufacturer. The RNA was incubated with 0.5 μg of oligo(dT)12–18mers primers (Invitrogen) for 7 min at 70 °C and then transferred onto ice. Then, 9 μL of a master mix containing 4 μL of SuperScript II buffer, 2 μL of 0.1 M DTT (Invitrogen), and 1 μL each of dNTPs (10 mM; Invitrogen), RNasin (40 UI; Promega, Madison, WI, USA) and SuperScript II (Invitrogen) were added, centrifuged and incubated at 42 °C for 60 min, followed by 5 min at 70 °C to inactivate the enzyme; the cDNA was stored at −20 °C.

### 3.4. Real-Time PCR Array Design and Testing

Most of the primers were obtained from the database of real-time primers curated by the Center for Medical Genetics (http://medgen.ugent.be/CMGG/). The remainders of the primers were designed using the online program Primer 3 (www.fokker.wi.mit.edu/primer3/input.htm). The primer selection parameters were primer size: 20–26 nts; primer melting temperature: 60 °C to 64 °C; GC clamp: 1; and product size range: generally 120–240 bp, but reduced to 100 bp if no appropriate primers could be identified. The sequences of the primers are listed in Supplementary File 1. All of the primers were synthesized by Invitrogen.

### 3.5. Real-Time PCR Array Analysis

Real-time PCR array analysis was performed in a total volume of 20 μL including 2 μL of cDNA, primers (0.2 mM each) and 10 μL of SYBR Green mix (Roche, Basel, Switzerland). Reactions were run on an Light cycler 480 (Roche, Basel, Switzerland) using universal thermal cycling parameters (95 °C for 5 min, 45 cycles of 10 s at 95 °C, 20 s at 60 °C and 15 s at 72 °C; followed by a melting curve: 10 s at 95 °C, 60 s at 60 °C and continued melting). The results were obtained using the sequence detection software of the Light cycler 480 and analyzed using Microsoft Excel. For quality control purposes, melting curves were acquired for all samples. The comparative C_t_ method was used to quantify gene expression. Firstly, the target gene expression level was normalized to expression of the housekeeping gene glyceraldehyde 3-phosphate dehydrogenase (*GAPDH*) within the same sample (−ΔCt), and then the relative expression of each gene was calculated using 10^6^ × Log_2_ (−ΔCt). The gene expression of the pediatric ALL and control samples was presented as the average ± SE.

### 3.6. Western Blot Analysis

For western blot analysis, cellular proteins were extracted in 40 mM Tris–HCl (pH 7.4) containing 150 mM NaCl and 1% (*v*/*v*) Triton X-100, supplemented with a cocktail of protease inhibitors. Equal amounts of protein were resolved on 12% SDS-PAGE gels, and then transferred to a PVDF membrane (Millipore, Bedford, MA, USA). Blots were blocked and then probed with antibodies against PAK1 (1:1000, Cell Signaling Technology, Inc., Danvers, MA, USA), HDAC2 (1:1000, Santa Cruz Biotechnology, Inc., Santa Cruz, CA, USA), GAPDH (1:5000, Sigma, St. Louis, MO, USA). After washing, the blots were incubated with horseradish peroxidase-conjugated secondary antibodies and visualized by enhanced chemiluminescence kit (Pierce, Rockford, IL, USA). Protein bands were visualized after exposure of the membrane to Kodak X-ray film.

### 3.7. Ingenuity Pathway Analysis (IPA)

Datasets derived from the real-time PCR array analyses, representing genes with significantly altered expression profiles, were imported into the Ingenuity Pathway Analysis Tool (IPA Tool; Ingenuity H Systems, Redwood City, CA, USA; http://www.ingenuity.com). In IPA, differentially expressed genes are mapped to the genetic networks available in the Ingenuity database, and then ranked by score. The Ingenuity Pathway Knowledge Base (IPKB) forms the basis of IPA, and is derived from known gene functions and interactions published in the literature. Thus, IPA enables the identification of biological networks, global functions and functional pathways for a particular dataset. The program also calculates the significance of the genes in the network, the other genes with which it interacts, and how the products of the genes directly or indirectly act on each other, including those not involved in the microarray analysis. The networks created are ranked, depending on the number of significantly expressed genes they contain, and also the list of significant, relevant diseases. A network is a graphical representation of the molecular relationships between molecules. Molecules are represented as nodes, and the biological relationship between two nodes is represented as an edge (line). All edges are supported by at least one reference from the literature, a textbook, or canonical information stored in the Ingenuity Pathways Knowledge Base. The intensity of the node color indicates the degree of upregulation (red) or downregulation (green). Nodes are displayed using various shapes that represent the functional class of the gene product.

### 3.8. Statistical Analysis

The significance of the differences in the gene expression profiles of pediatric ALL and the control samples were calculated using unpaired *t*-tests with SPSS version 11.5 (SPSS Inc., Chicago, IL, USA); *p* values <0.05 were considered statistically significant.

## 4. Conclusions

We successfully designed and tested a real-time PCR array for analysis of the genes encoding human epigenetic chromatin modification enzymes. Using this array, we demonstrated the different mRNA expression patterns of human histone-modifying enzymes in normal karyotype B cell pediatric ALL and normal controls. A lot of genes can be significantly clustered in the gene and sample analysis, including the histone deacetylases *HDAC2* which was upregulated in normal karyotype B cell pediatric ALL, *PRMT2* and the putative tumor suppressor gene *EP300* which were downregulated in pediatric ALL. We identified a number of dysregulated histone-modifying enzymes in normal karyotype B cell pediatric ALL, which have not previously been reported to be differently expressed in pediatric ALL. Future studies will seek to determine whether these dys-regulated histone-modifying enzymes can serve as biomarkers of pediatric ALL. Additionally, IPA indicated that curcumin and miR-34 may be the major upstream regulators of histone-modifying enzymes in normal karyotype B cell pediatric ALL, future studies will seek to validate these results, and examine the role of curcumin and miR-34 in the molecular basis of leukemia. This work provides new clues regarding the molecular mechanisms which regulate the development of normal karyotype B cell pediatric ALL.

## Figures and Tables

**Table 1:** Clinical features of the normal donors and pediatric acute lymphoblastic leukemia patients.

		NBM/ITP	Pediatric ALL
Age		4.3 (0.7–13.6)	5.1 (0.9–13.6)
Sex (M/F)		12/8	19/11
White blood cells (109/L)		8.4 (3.82–16.97)	56.9 (2.1–638)
Hemoglobin (g/L)		129 (90–157)	81.2 (28–126)
Platelet count (109/L)		313 (17–498)	49 (8–195)
Immunophenotyping	B-ALL	ns	28
	T-ALL	ns	2
Risk stratification	Standard	ns	6
	Median	ns	8
	High	ns	16
Karyotype	Normal	ns	18
	Abnormal	ns	12
Fusion gene	MLL	ns	2
	TEL/AML1	ns	7
	BCR/ABL1	ns	1
	E2A/PBX	ns	1

NBM, Normal Bone Marrow; IPT, Idiopathic Thrombocytopenic Purpura; B-ALL, B cell acute lymphoblastic leukemia; T-ALL, T cell acute lymphoblastic leukemia

**Table 2:** Genes encoding epigenetic chromatin modification enzymes upregulated in normal karyotype B cell pediatric ALL compared with normal controls.

	Gene	Description	NBM	ALL	Change	*p* value
1	PAK1	P21 protein (Cdc42/Rac)-activated kinase 1	690.78	8684.84	12.57	3.94 × 10^−17^
2	EHMT2	Euchromatic histone-lysine *N*-methyltransferase 2	1238.91	19701.33	15.90	1.07 × 10^−15^
3	KAT7	K(lysine) acetyltransferase 7	13037.87	76644.47	5.88	5.97 × 10^−9^
4	GCN5L2	K(lysine) acetyltransferase 2A	6554.99	33808.15	5.16	6.03 × 10^−9^
5	SUZ12	Suppressor of zeste 12 homolog	24556.24	123398.3	5.03	1.56 × 10^−6^
6	SUV420H1	Suppressor of variegation 4–20 homolog 1	7843.03	44187.54	5.63	1.65 × 10^−6^
7	KAT6B	K(lysine) acetyltransferase 6B	26130.51	75299.71	2.88	4.19 × 10^−6^
8	CSRP2BP	CSRP2 binding protein	2041.52	14595.22	7.15	2.25 × 10^−5^
9	RNF20	Ring finger protein 20	10498.77	32675.14	3.11	0.00034
10	SETD2	SET domain containing 2	14027.6	42467.29	3.02	0.008
11	HDAC2	Histone deacetylase 2	9325.25	50147.01	5.38	0.015

NBM, Normal Bone Marrow.

**Table 3:** Genes encoding epigenetic chromatin modification enzymes downregulated in normal karyotype B cell pediatric ALL compared with normal controls.

	Gene	Description	NBM	ALL	Change	*p* value
1	HDAC5	Histone deacetylase 5	11379.83	186.94	0.01	2.67 × 10^−27^
2	NOTCH2	Notch homolog 2	32473.8	319.09	0.01	3.05 × 10^−27^
3	NOTCH1	Notch homolog 1	13109.41	1089.23	0.05	9.19 × 10^−15^
4	EP300	E1A binding protein p300	47487.19	5601.60	0.12	1.37 × 10^−13^
5	PRMT2	Protein arginine methyltransferase 2	28388.11	6336.82	0.22	2.08 × 10^−12^
6	DNMT3A	DNA (cytosine) methyltransferase 3 alpha	3868.084	682.15	0.17	2.04 × 10^−12^
7	RPS6KA3	Ribosomal protein S6 polypeptide 3	20389.11	5203.03	0.25	1.00 × 10^−5^

NBM, Normal Bone Marrow.

## Supplementary Files

Supplementary File 1 Supplemental Table (XLS, 34 KB)

Supplementary File 2 Supplemental Graphic (JPG, 1468 KB)

Supplementary File 3 Supplementary (PDF, 298 KB)
