# Supplementary figures and images for: Differential mRNA Expression Levels of Human Histone-Modifying Enzymes in Normal Karyotype B Cell Pediatric Acute Lymphoblastic Leukemia
Source: Int J Mol Sci. 2013 Feb 6;14(2):3376–94. doi: 10.3390/ijms14023376 (PMC3588049; doi:10.3390/ijms14023376)

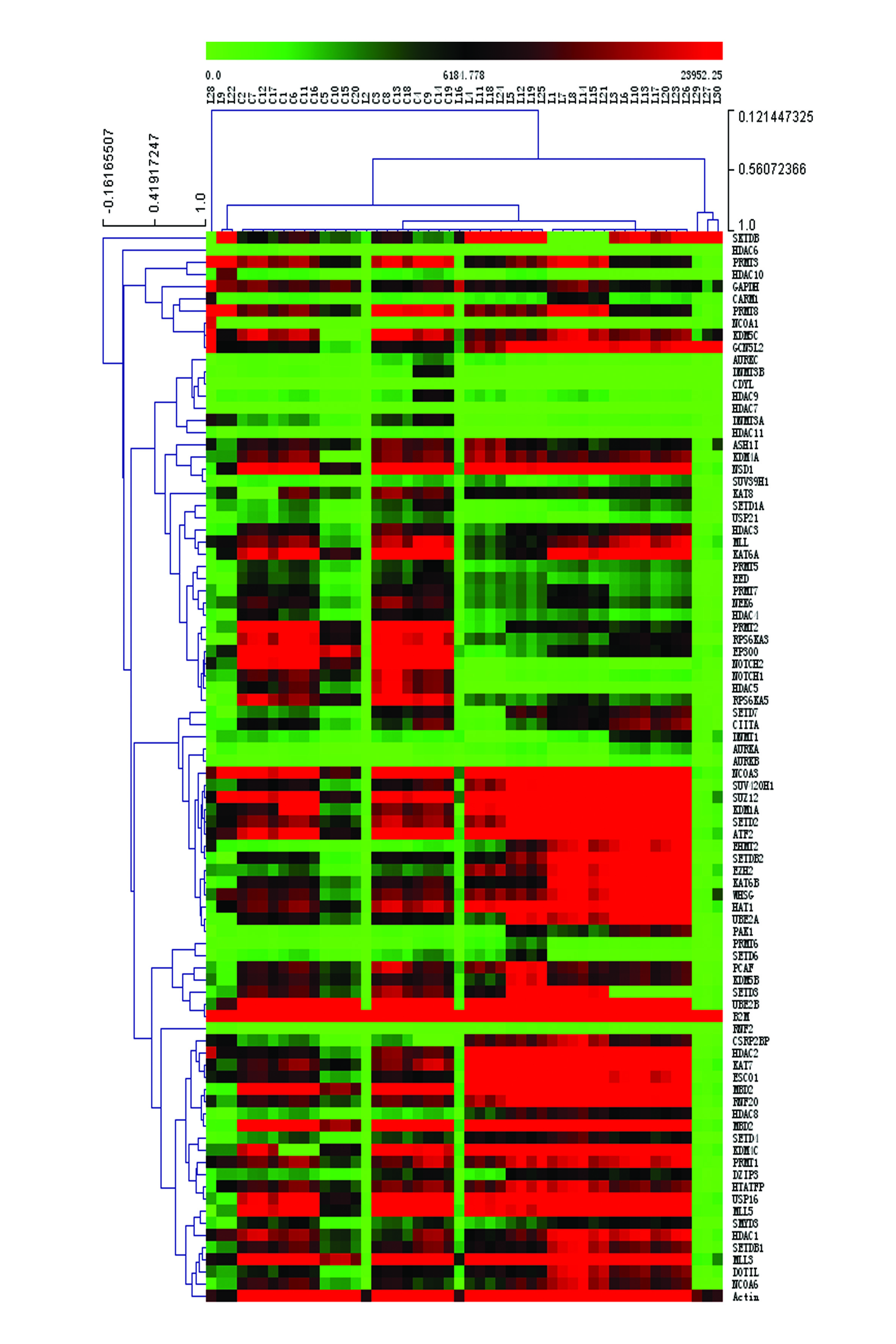

Supplement: Supplementary File 2 — Supplemental Graphic (JPG, 1468 KB) [file ijms-14-03376-s002.jpg]
