# Supplementary material for: Differential mRNA Expression Levels of Human Histone-Modifying Enzymes in Normal Karyotype B Cell Pediatric Acute Lymphoblastic Leukemia
Source: Int J Mol Sci. 2013 Feb 6;14(2):3376–94. doi: 10.3390/ijms14023376 (PMC3588049; doi:10.3390/ijms14023376)
Supplement: Supplementary File 3 — Supplementary (PDF, 298 KB) [file ijms-14-03376-s003.pdf]

Analysis Name: genes for IPA - 2013-01-05 12:52
Analysis Creation Date: 2013-01-05
Build version: 192063
Content version: 14400082 (Release Date: 2012-11-01)

## Analysis settings

View
Reference set: Ingenuity Knowledge Base (Genes Only)
Relationship to include: Direct and Indirect
Includes Endogenous Chemicals
Optional Analyses: My Pathways My List

Filter Summary:
Consider only molecules and/or relationships where
(species = Uncategorized OR Human) AND
(confidence = Experimentally Observed) AND
(tissues/cell lines = Cytotoxic T cells OR Activated CD56bright NK cells OR Salivary Gland OR Immature monocyte-derived dendritic cells OR Lung OR Central memory cytotoxic T cells OR Large Intestine OR K-562 OR MOLT-4 OR CD56bright NK cells OR Macrophages OR BDCA-1+ dendritic cells OR Kidney OR Prostate Gland OR Testis OR SR OR Effector memory cytotoxic T cells OR Bladder OR Activated Vd2 Gamma-delta T cells OR Mammary Gland OR Naive B cells OR CCRF-CEM OR Activated Vd1 Gamma-delta T cells OR Central memory helper T cells OR Skeletal Muscle OR Liver OR Epidermis OR Ovary OR Naive helper T cells OR Jurkat OR RAW 264.7 OR Activated helper T cells OR Heart OR Small Intestine OR BDCA-3+ dendritic cells OR Thymus OR Uterus OR Murine NKT cells OR Plasmacytoid dendritic cells OR Other Tissues and Primary Cells OR Stomach OR Memory B cells OR Monocytes OR Retina OR Vd2 Gamma-delta T cells OR Effector memory helper T cells OR Mature monocyte-derived dendritic cells OR Neutrophils OR Effector memory RA+ cytotoxic T cells OR Pancreas OR Th2 cells OR Spleen

(c) 2000-2013 Ingenuity Systems, Inc. All rights reserved.

OR Adipose OR HL-60 OR Activated CD56dim NK cells OR Placenta OR Vd1 Gamma-delta T cells OR THP-1 OR Natural T-regulatory cells OR Th1 cells OR Monocyte-derived macrophage OR Effector T cells OR CD56dim NK cells) AND
(data sources = BIND OR BIOGRID OR Cognia OR DIP OR Ingenuity Expert Findings OR Ingenuity ExpertAssist Findings OR INTACT OR Interactome studies OR MINT OR MIPS OR TarBase)

## Top Networks

| ID | Associated Network Functions | Score |
|---|---|---|
| 1 | Gene Expression, Organ Morphology, Skeletal and Muscular System Development and Function | 35 |
| 2 | DNA Replication, Recombination, and Repair, Gene Expression, Cellular Function and Maintenance | 10 |

(c) 2000-2013 Ingenuity Systems, Inc. All rights reserved.

INGENUITY® SYSTEMS

## Top Bio Functions

### Diseases and Disorders

| Name | p-value | # Molecules |
|---|---|---|
| Cancer | 5.42E-06 - 4.58E-02 | 12 |
| Hematological Disease | 5.42E-06 - 1.93E-02 | 8 |
| Immunological Disease | 8.31E-05 - 1.04E-02 | 6 |
| Developmental Disorder | 1.15E-03 - 4.33E-02 | 5 |
| Hereditary Disorder | 1.15E-03 - 2.82E-02 | 5 |

### Molecular and Cellular Functions

| Name | p-value | # Molecules |
|---|---|---|
| Gene Expression | 5.46E-09 - 2.06E-02 | 13 |
| Cell Cycle | 2.79E-06 - 2.57E-02 | 5 |
| Cellular Development | 2.09E-05 - 4.08E-02 | 7 |
| Cellular Growth and Proliferation | 2.09E-05 - 4.08E-02 | 7 |
| Cell Death and Survival | 1.70E-04 - 3.64E-02 | 8 |

### Physiological System Development and Function

| Name | p-value | # Molecules |
|---|---|---|
| Cardiovascular System Development and Function | 2.38E-05 - 5.19E-03 | 2 |
| Embryonic Development | 2.38E-05 - 2.95E-02 | 4 |
| Organ Development | 2.38E-05 - 5.19E-03 | 3 |
| Organismal Development | 2.38E-05 - 5.19E-03 | 3 |
| Tissue Development | 2.38E-05 - 1.16E-02 | 5 |

(c) 2000-2013 Ingenuity Systems, Inc. All rights reserved.

INGENUITY SYSTEMS

## Top Canonical Pathways

| Name | p-value | Ratio |
|---|---|---|
| Phospholipase C Signaling | 1.79E-04 | 4/256 (0.016) |
| HMGB1 Signaling | 2.22E-04 | 3/99 (0.03) |
| DNA Methylation and Transcriptional Repression Signaling | 2.99E-04 | 2/23 (0.087) |
| Hereditary Breast Cancer Signaling | 3.84E-04 | 3/123 (0.024) |
| Notch Signaling | 1.03E-03 | 2/41 (0.049) |

## Top Molecules

### Fold Change up-regulated

| Molecules | Exp. Value | Exp. Chart |
|---|---|---|
| CSRP2BP | 1.000 | |
| EHMT2 | 1.000 | |
| HDAC2 | 1.000 | |
| KAT7 | 1.000 | |
| KAT2A | 1.000 | |
| KAT6B | 1.000 | |
| PAK1 | 1.000 | |
| RNF20 | 1.000 | |
| SETD2 | 1.000 | |
| SUV420H1 | 1.000 | |

### Fold Change down-regulated

| Molecules | Exp. Value | Exp. Chart |
|---|---|---|
| RPS6KA3 | -1.000 | |
| PRMT2 | -1.000 | |
| NOTCH2 | -1.000 | |

(c) 2000-2013 Ingenuity Systems, Inc. All rights reserved.

| | |
|---|---|
| NOTCH1 | -1.000 |
| HDAC5 | -1.000 |
| EP300 | -1.000 |
| DNMT3A | -1.000 |

## Top Upstream Regulators

| Upstream Regulator | p-value of overlap | Predicted Activation State |
|---|---|---|
| curcumin | 2.83E-06 | |
| mir-34 | 2.45E-05 | |
| miR-34a-5p (and other miRNAs w/seed GGCAGUG) | 1.33E-04 | |
| POFUT1 | 9.63E-04 | |
| PCNA | 9.63E-04 | |

(c) 2000-2013 Ingenuity Systems, Inc. All rights reserved.

## Top My Lists

| Name | p-value | Ratio |
|---|---|---|

## Top My Pathways

| Name | p-value | Ratio |
|---|---|---|
| Merged networks | 6.93E-02 | 4/1404 (0.003) |
| FASN malignancy pathway | 1.1E-01 | 1/142 (0.007) |
| NRF2-mediated Oxidative Stress Response | 2.05E-01 | 1/194 (0.005) |

## Top Tox Lists

| Name | p-value | Ratio |
|---|---|---|
| Cell Cycle: G1/S Checkpoint Regulation | 2.7E-03 | 2/60 (0.033) |
| Cardiac Hypertrophy | 8.11E-03 | 3/326 (0.009) |
| RAR Activation | 2.04E-02 | 2/171 (0.012) |
| NF-κB Signaling | 2.08E-02 | 2/173 (0.012) |
| Increases Cardiac Dilation | 3.45E-02 | 1/27 (0.037) |

(c) 2000-2013 Ingenuity Systems, Inc. All rights reserved.

INGENUITY® SYSTEMS

## Top Tox Functions

### Cardiotoxicity

| Name | p-value | # Molecules |
|---|---|---|
| Cardiac Hypertrophy | 2.27E-03 - 4.33E-02 | 2 |
| Heart Failure | 1.55E-01 - 1.55E-01 | 1 |

### Hepatotoxicity

| Name | p-value | # Molecules |
|---|---|---|
| Liver Cirrhosis | 5.46E-03 - 5.46E-03 | 2 |
| Hepatocellular Carcinoma | 4.97E-01 - 4.97E-01 | 1 |
| Liver Hyperplasia/Hyperproliferation | 4.97E-01 - 4.97E-01 | 1 |

### Nephrotoxicity

| Name | p-value | # Molecules |
|---|---|---|
| Renal Necrosis/Cell Death | 3.64E-02 - 6.79E-02 | 2 |

(c) 2000-2013 Ingenuity Systems, Inc. All rights reserved.

INGENUITY® SYSTEMS
